# Nano-hydroxyapatite-assisted enzyme-induced carbonate precipitation enhances Pb-contaminated aqueous solution and loess remediation

**DOI:** 10.3389/fbioe.2024.1410203

**Published:** 2024-06-27

**Authors:** Zhao-Wei Bian, Wen-Chieh Cheng, Yi-Xin Xie, Md Mizanur Rahman, Wenjie He

**Affiliations:** ^1^ School of Civil Engineering, Xi’an University of Architecture and Technology, Xi’an, China; ^2^ Shaanxi Jianke Construction Special Engineering Co., Ltd., Xi’an, China; ^3^ Shaanxi Key Laboratory of Geotechnical and Underground Space Engineering (XAUAT), Xi’an, China; ^4^ UniSA STEM, ScaRCE, University of South Australia, Adelaide, SA, Australia

**Keywords:** urea hydrolysis, lead, nano-hydroxyapatite, cerussite, carbonate-bearing hydroxylpyromorphite

## Abstract

Intensive agricultural activities could cause lead (Pb) bioaccumulation, threatening human health. Although the enzyme-induced carbonate precipitation (EICP) technology has been applied to tackle the aforesaid problem, the urease may denature or even lose its activity when subjected to a significant Pb^2+^ toxicity effect. To this end, the nano-hydroxyapatite (nHAP)-assisted EICP was proposed to reduce the mobility of Pb^2+^. Results indicated that a below 30% immobilization efficiency at 60 mM Pb^2+^ was attained under EICP. nHAP adsorbed the majority of Pb^2+^, preventing Pb^2+^ attachment to urease. Further, hydroxylphosphohedyphane or hydroxylpyromorphite was formed at 60 mM Pb^2+^, followed by the formation of cerussite, allowing hydroxylphosphohedyphane or hydroxylpyromorphite to be wrapped by cerussite. By contrast, carbonate-bearing hydroxylpyromorphite of higher stability (Pb_10_(PO_4_)_6_CO_3_) was developed at 20 mM Pb^2+^ as CO_3_^2−^ substituted the hydroxyl group in hydroxylpyromorphite. Moreover, nHAP helped EICP to form nucleated minerals. As a result, the EICP-nHAP technology raised the immobilization efficiency at 60 mM Pb^2+^ up to 70%. The findings highlight the potential of applying the EICP-nHAP technology to Pb-containing water bodies remediation.

## 1 Introduction

Although intensive agricultural activities lead to a good crop, heavy metal could build up in the body during a chronic exposure (Karaouzas et al., 2020; Chen et al., 2024a; Chen et al., 2024b). Lead (Pb) poisoning, also known as plumbism and saturnism, is identified as blood lead level surpassing the upper limit of 10 μg/100 g set by the United States Centers for Disease Control and Prevention. It has symptoms predominantly in the central nervous system. The symptoms like loss of appetite, memory loss, and kidney failure develop over weeks to months, but acute symptoms from intensive exposures also occur (Bai et al., 2021; Xu et al., 2023; Liu et al., 2024; Xue et al., 2024a; Wang et al., 2024a). In severe instances, seizures, coma, or death may take place (Costa de Almeida et al., 2007; Chen et al., 2021). To tackle the said issue, many remediation technologies applied for Pb immobilization have been developed (Bai et al., 2022; Xie et al., 2022; Xue et al., 2022; Xue et al., 2023). However, they are often criticized due to poor performance and are accompanied by a high risk of secondary contamination (Yang et al., 2020; Meng et al., 2022; Hu et al., 2023a; Hu et al., 2023b; Wang et al., 2023; Bai et al., 2024; Xue et al., 2024b).

Enzyme-induced carbonate precipitation (EICP) has recently drawn particular attention as it featured high manoeuvrability and low risk of secondary pollution. Urease catalyzes the hydrolysis of urea resulting in the production of carbonate ions and the rise of surrounding pH. Then precipitation of CaCO_3_ in between soil particles enhances shear strength and erosion resistance of soil. The urease of a nanometer in size allows for catalyzing urea hydrolysis in deep grounds (Ashkan et al., 2019; Gowthaman et al., 2021; Gowthaman et al., 2023). Previous studies concentrate on problems of low pollution concentration (<5 mM Pb2+ in water bodies or < 400 mg/kg Pb^2+^ in soils) (Qian et al., 2017; Peng et al., 2020). However, the reality is far worse than people imagined (Chen et al., 2023). For instance, lead-containing wastewater with a Pb^2+^ concentration of 147 mM was found close to a lead-zinc smelting plant in Southeast China (Duan et al., 2016).

Fang et al. (2021) reported that Ca^2+^ addition enhanced bacterial tolerance to Cd^2+^ through competitive adsorption. The minimum inhibition concentration may affect Pb immobilization, although the initial urease activity (UA) was positively correlated to bacterial tolerance (Jiang et al., 2019; Duarte-Nass et al., 2020). Nano-hydroxyapatite (nHAP), with a chemical formula Ca_10_(PO_4_)_6_(OH)_2_, has a strong potential to work with EICP as it features a large specific surface area and high solubility (Xie et al., 2024; Wang et al., 2024). Its combination with heavy metal ions is as expected (Javadinejad et al., 2017). However, a large body of research does not pay attention to the pathways applied to Pb immobilization, how the nHAP application changes the nucleation and crystallization of bioprecipitates, and the interplay with Ca^2+^ application. The above accentuates several research gaps and shortcomings that remain to be addressed. The main objectives of this study are to: (1) investigate the role of nHAP in preventing Pb^2+^ attachment; and (2) explore the inherent mechanism that affects Pb immobilization.

## 2 Materials and methods

### 2.1 Urease extraction and nano-hydroxyapatite

This study tested the tolerance of urease to 20 mM–60 mM Pb2^+^ (Duan et al., 2016; Jiang et al., 2019). Then the results were compared to those that neglected the nHAP application to highlight the relative merits of the nHAP application. Apart from that, the enhancement of Pb immobilization would not have been achieved if the inherence mechanism had not been explored. To this end, a series of samples taken from the bioprecipitates were characterized through SEM, SEM-EDS, FTIR, and XRD. These provide details about the design of experiments and the logic behind this study.

This study first extracted crude urease from jack beans (*Canavalia ensiformis* L.). After crushing and sieving the dried jack beans, the bean powder was yielded. It helped prepare a stock solution stored at minus four degrees Celsius. Then the stock solution was centrifuged allowing the supernatant to be extracted as the urease solution. Pb^2+^ attachment could cause the urease to lose its activity, leading to an inability to secure the degree of urea hydrolysis and immobilization efficiency. This study attempted to tackle the said problem by introducing nHAP. nHAP is originally a natural apatite mineral. It could also be synthetic nowadays as a result of the strong demand in bone tissue engineering (Qiu et al., 2021). Its large specific surface area creates an ideal environment for combination with heavy metal ions. nHAP, acquired from Shanghai Macklin Biochemical Co., Ltd, consisted of hydroxyapatite nanoparticles of 60–80 nm in average size and assisted EICP in reducing the mobility of Pb^2+^, which is referred to also as ‘EICP-nHAP’.

### 2.2 Characteristics of urease

UA corresponds to the rate of urea hydrolysis. In the present work, UA was measured via a method suggested by Whiffin et al. (2007), in which 1 mL urease solution is added to 9 mL urea solution at 1.11 M, and the resultant change in electric conductivity (EC) over a 5-min period at 20°C ± 2°C was applied to determine UA by Eq. 1.

$$UA=\frac{{EC}_{5}-{EC}_{0}}{5}\times 10\times 1.11\;\left(\text{mM Urea }/\;\min\right)$$ (1)

where *EC*_0_ and *EC*_5_ are identical to EC at 0 and 5 min, respectively.

### 2.3 Pb immobilization

EICP as a basis for comparison was applied to the present work when subjected to 20–60 mM Pb2^+^. Pb-containing solutions were prepared using reagents consisting of 100 mM urea and lead nitrate. Then a 5% urease solution was inoculated into a 100 mLPb-containing solution to catalyze urea hydrolysis toward achieving Pb immobilization. This process remained for a 48-h period. Once the remaining Pb^2+^ concentration was measured, the immobilization efficiency can be evaluated through Eq. 2:

$$\text{Immobilization efficiency}=\frac{C_{0}-C_{1}}{C_{0}}\times 100\%$$ (2)

where *C*_0_ and *C*_1_ represent the initial and remaining Pb^2+^ concentrations, respectively. To highlight the relative merits of EICP-nHAP, 100 mg nHAP was appended to the Pb-contaminated solution at the beginning. The remaining steps applied to catalyze urea hydrolysis were the same as in EICP. Then a comparison in the immobilization efficiency between EICP and EICP-nHAP not only enhanced our understanding of the relative merits of EICP-nHAP but also explored the potential of applying EICP-nHAP to Pb-containing water bodies remediation. Apart from that, NH_4_^+^ concentration, surrounding pH, and Pb^2+^ concentration were recorded per the given frequencies. A Nessler spectrophotometer, a pH meter (HI2003; Hanna Instruments Inc., Italy), and an atomic adsorption spectrophotometer (TAS-990; Beijing Purkinje General Instrument Co., Ltd., China) helped to accomplish the measurements.

On the other hand, one-dimensional soil column tests were conducted to see how Pb species affected Pb immobilization. To this end, a series of block loess samples were taken from northwest China. The loess contained 14.2% sand and 85.8% fines content. Its liquid limit (LL) and plasticity index (PI) were 24.4%, and 10.2%, respectively. It was classified as low plasticity clay (CL) using the Unified Soil Classification System (USCS) (ASTM D2487-17, 2010). A mixture of Pb-contaminated loess and nHAP was loaded into the one-dimensional columnar container of 180 mm in height and 61.8 mm in diameter applied to the one-dimensional soil column tests. Three Pb^2+^ concentrations, namely, 4,000 mg/kg, 8,000 mg/kg, and 16,000 mg/kg, were chosen. After that, the urea solution was injected at 5 mL/min into the Pb-contaminated loess. A control group (CG) that neglected both EICP and EICP-nHAP was set up for comparison sake. The samples taken after 24 h were analyzed based on Tessier sequential extraction procedure.

### 2.4 Sample characterization

The bioprecipitates were extracted following the EICP (or EICP-nHAP) process, preparing samples using the freeze-drying method. The frozen temperature was set at −5°C. The frozen samples were then stood still at −50°C. SEM, SEM-EDS, FTIR, and XRD were applied in their characterization. The SEM and SEM-EDS images were acquired from a scanning electron microscope (Sigma 300; ZEISS, Germany). The chemical bonds and surface functional groups (e.g., hydroxyl and carboxyl groups) involved in Pb immobilization were analyzed via a Fourier transform infrared spectrometer (Nicolet iS50; Thermo Scientific, United States of America). While the mineralogical compositions of the samples were identified using an X-ray diffractometer (D8 Advance; Bruker AXS, Germany) where the 2θ varied within a range of 5°–85°, with a step width of 0.02° and a scanning rate of 2°/min. MDI Jade software (version 9.5) was responsible for XRD pattern processing.

Five Pb species in Tessier sequential extraction procedure were measured by an atomic absorption spectrometer (TAS-990; Persee Inc., China) where EXC denoted Exchangeable state-Pb, CAR denoted Carbonate combination state-Pb, OX denoted Fe–Mn oxides state-Pb, ORG denoted Organic state-Pb, and RES denoted Residue state-Pb. Three replicates were applied to each of the tests to verify the significance of the results, and the error bars on a graph were used to help see the margins of error and standard deviations at a glance.

## 3 Results and discussion

### 3.1 Efficiency deterioration

**FIGURE 1:**
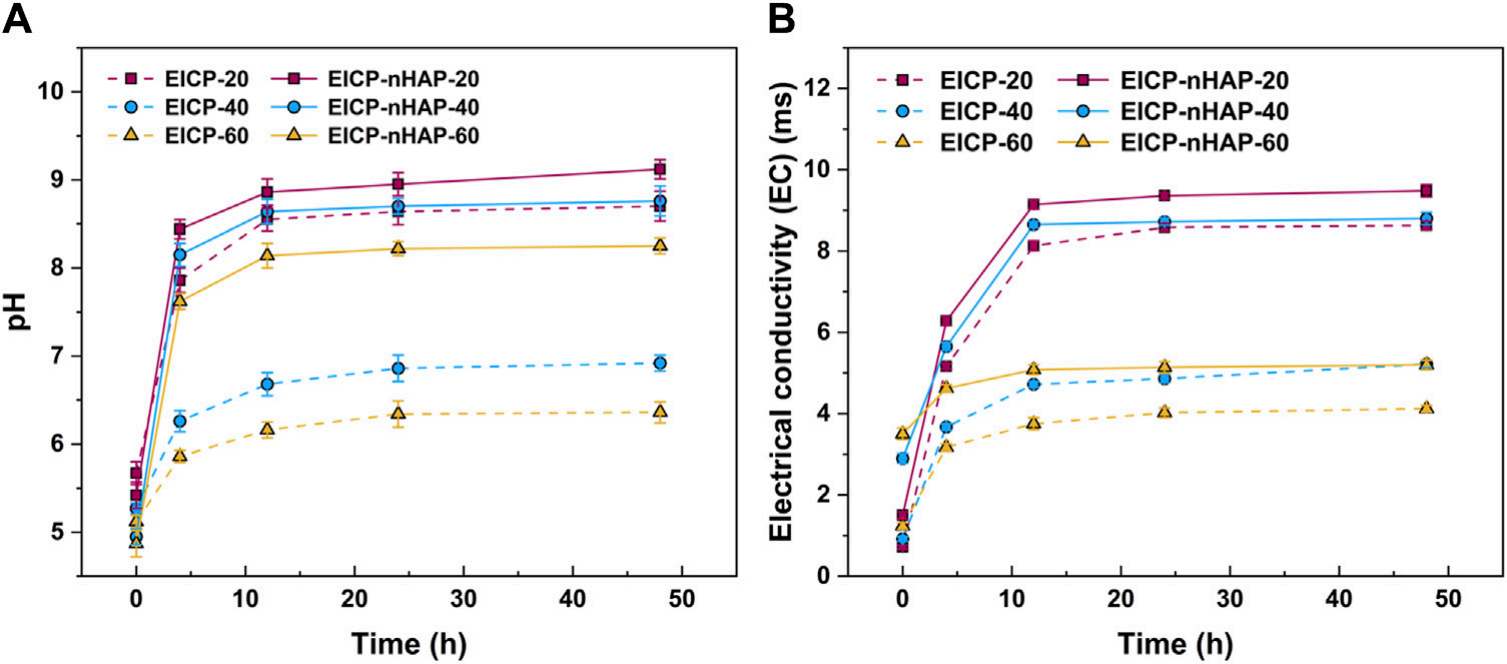
Temporal relationships of **(A)** pH and **(B)** EC applied to EICP and EICP-nHAP when subjected to 20 mM, 40 mM, and 60 mM Pb^2+^.

**FIGURE 2:**
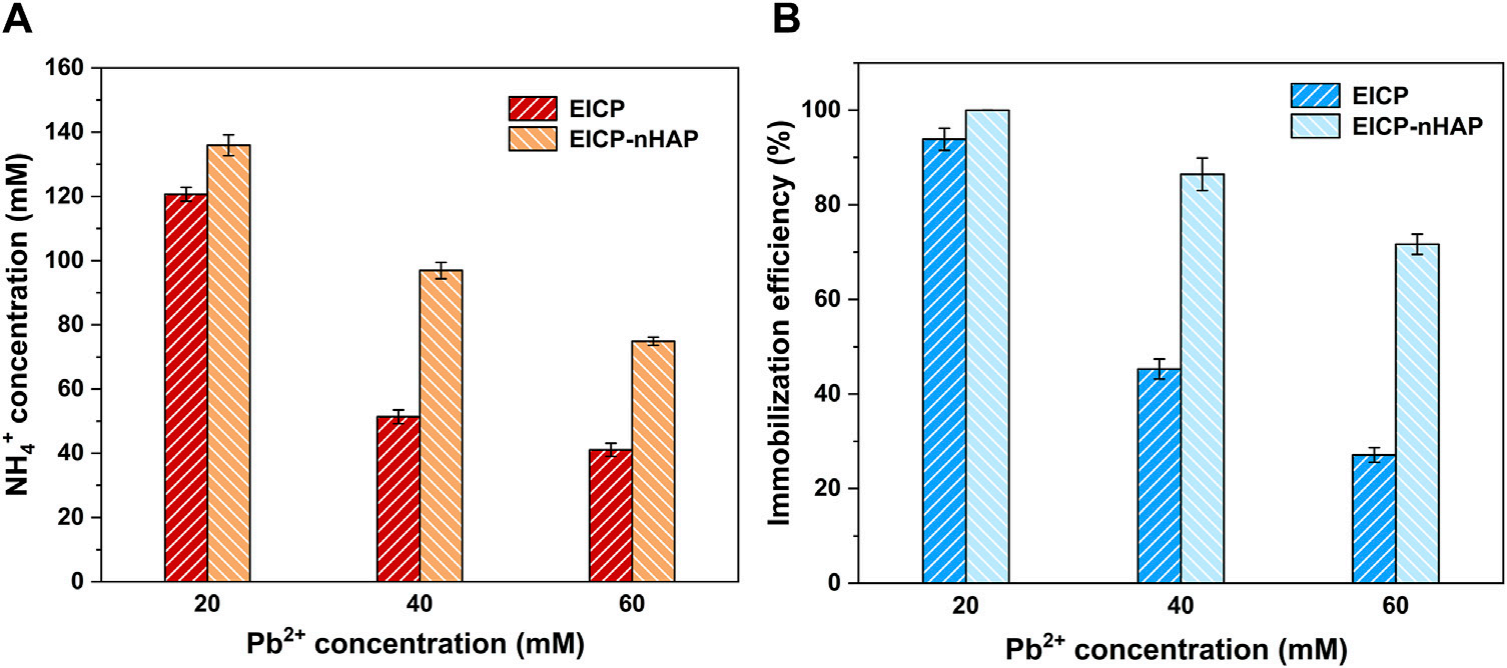
**(A)** Relationships of NH_4_^+^ concentration versus Pb^2+^ concentration applied to EICP and EICP-nHAP and **(B)** relationships of immobilization efficiency versus Pb^2+^ concentration applied to EICP and EICP-nHAP.

The temporal relationships of pH and electric conductivity (EC) under EICP and EICP-nHAP are shown in Figure 1A. Under EICP, pH, when subjected to 20 mM Pb2^+^, increased sharply and then reached approximately 8.5 at 48 h after the hydrolysis of urea. However, pH increased at a slower pace and then reached 6.9 at 48 h when subjected to 40 mM Pb^2+^, indicating the effect of Pb^2+^ toxicity. The most significant effect of Pb^2+^ toxicity was present at 60 mM Pb^2+^. This led to an inability for the urease to catalyze urea hydrolysis, leading to a pH of below 6.5 at 48 h. The temporal relationships of EC are shown in Figure 1B. Similarly, the value of EC went down when Pb^2+^ concentration went up. The difference in EC between 20 mM Pb^2+^ and 60 mM Pb^2+^ reached 4.5 m. The variations of NH_4_^+^ concentration against Pb^2+^ concentration are depicted in Figure 2A. The more significant the effect of Pb^2+^ toxicity, the lower the NH_4_^+^ concentration, and the lower the degree of urea hydrolysis. These results led us to summarize that the urease lost its activity when Pb^2+^ concentration exceeded 20 mL/L and that the immobilization efficiency dropped to a value as low as about 25% as a result of a reduction in the degree of urea hydrolysis (see Figure 2B).

### 3.2 Pb immobilization enhancement

By contrast, under EICP-nHAP, pH reached 9 and 8.3 at 48 h when subjected to 20 mM Pb^2+^ and 60 mM Pb^2+^, respectively (see Figure 1A). They were higher than those under EICP. EC was also higher under EICP-nHAP (see Figure 1B). The difference in EC between EICP and EICP-nHAP increased with increasing Pb^2+^ concentration. The difference in NH_4_^+^ between EICP and EICP-nHAP increased from 15 mM at 20 mM Pb^2+^ to 35 mM at 60 mM Pb^2+^ (see Figure 2A). The variations of immobilization efficiency against Pb^2+^ concentration are depicted in Figure 2B. The difference in immobilization efficiency between EICP and EICP-nHAP increased from 5% at 20 mM Pb^2+^ to 45% at 60 mM Pb^2+^. These results indicated that nHAP application prevented Pb^2+^ attachment and allowed UA and the degree of urea hydrolysis to remain high enough. As a result, the immobilization efficiency reached above 70% when even subjected to 60 mM Pb^2+^.

### 3.3 Sample characterization

**FIGURE 3:**
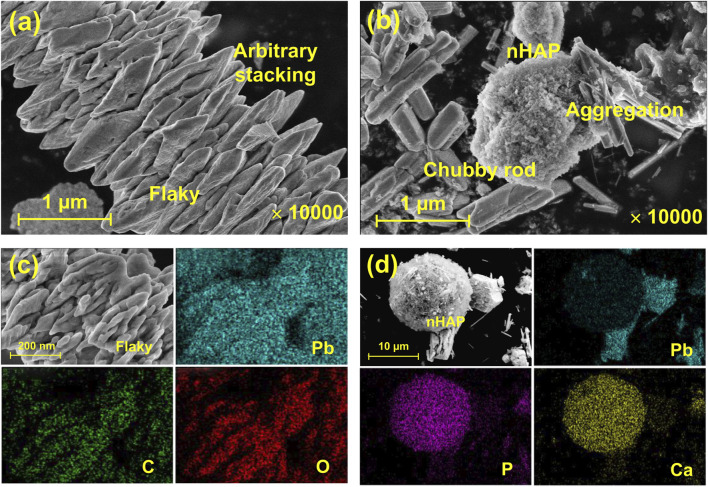
SEM test results applied to **(A)** EICP (x 10,000) and **(B)** EICP-nHAP (x 10,000); SEM-EDS test results applied to **(C)** EICP (x 2000) and **(D)** EICP-nHAP (x 1,000).

Results from the SEM images showed that under EICP, non-nucleated, flaky minerals were formed as a result of the inability of urease to provide nucleation sites (Figures 3A, B). By contrast, nucleated, chubby rod-shaped minerals were precipitated under EICP-nHAP. Compared to the non-nucleated minerals, the nucleated minerals featured higher stability since nHAP addressed the inability to provide nucleation sites by the urease (Heidarzadeh et al., 2019). This also explained that the nucleated minerals were not precipitated arbitrarily but directionally. On the other hand, C and O elements were distributed uniformly in the SEM-EDS images, meaning that under EICP, carbonate minerals were considered the main contributor to Pb immobilization (Figures 3C, D). Ca and P elements were present in the SEM-EDS images, implying that under EICP-nHAP, hydroxyapatite ruled Pb immobilization.

**FIGURE 4:**
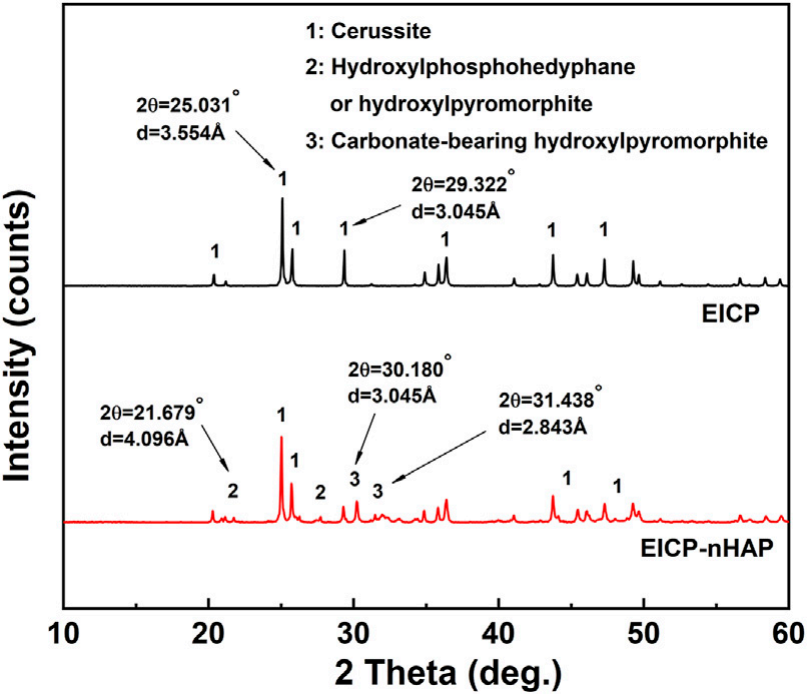
XRD spectrum applied to EICP and EICP-nHAP.

**FIGURE 5:**
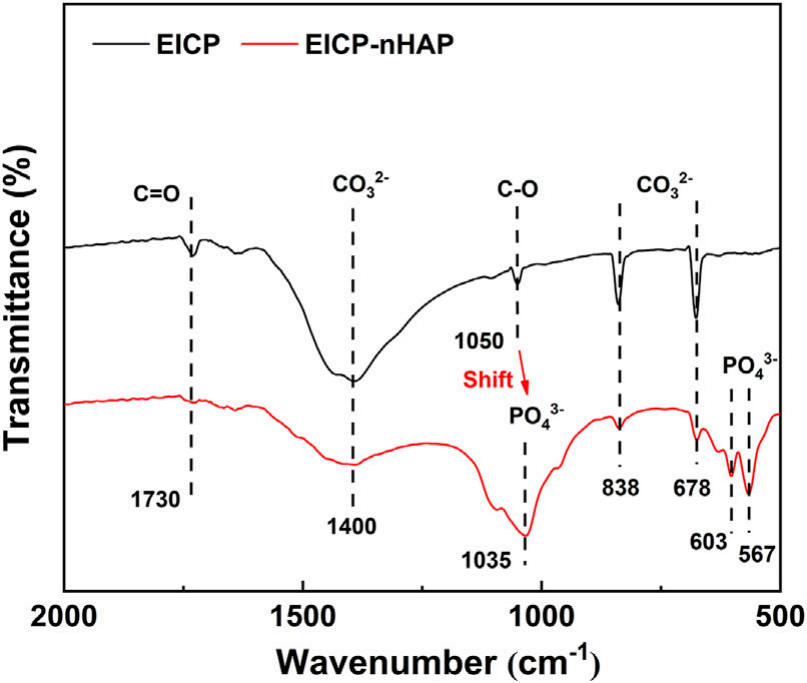
FTIR spectrum applied to EICP and EICP-nHAP.

Under EICP, the diffraction peak at 3.554 Å was ascribed to forming cerussite (PbCO_3_), although its formation was also related to other diffraction peaks of relatively weak intensity at 3.054 Å (Figure 4). Hydroxylphosphohedyphane (Ca_10-x_Pb_x_(PO_4_)_6_(OH)_2_) and hydroxylpyromorphite (Pb_10_(PO_4_)_6_(OH)_2_) were present under EICP-nHAP. On the other hand, the adsorption bands were specified at 1730 cm^−1^ and 1,050 cm^−1^, which were conformable to C = O and C-O stretching vibrations, respectively (Figure 5) (Bao et al., 2008; Timilsena et al., 2015; Ren et al., 2022). Carbonates were characterized by strong bands at 1,400 cm^−1^ (C-O bond antisymmetric stretch) and in the 678–838 cm^−1^ (CO_3_^2-^ deformation) region. These results provided eloquent testimony to support the above claim that carbonate minerals were the main contributor to Pb immobilization under EICP. Under EICP-nHAP, the nHAP application caused the band to shift to 1,035 cm^−1^ from 1,050 cm^−1^. Phosphates were also specified by strong adsorption bands within the 567–603 cm^−1^ (P-O stretch) region. These results were ascribed to the presence of PO_4_^3−^, indicating that hydroxyapatite primarily governed Pb immobilization under EICP-nHAP (Gao et al., 2019).

**FIGURE 6:**
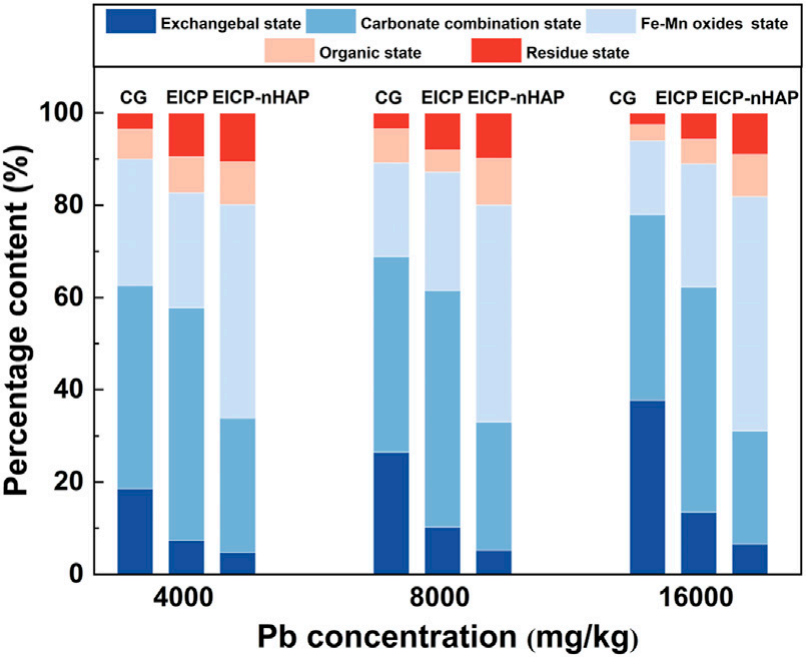
Distributions of Pb species versus Pb concentration applied to CG, EICP, and EICP-nHAP.

CAR-Pb was the major species (approximately 50%) under EICP, while OX-Pb was the major species (about 46.7%) under EICP-nHAP (see Figure 6). In addition to transforming CAR-Pb into OX-Pb, EICP-nHAP reduced the fraction of EXC-Pb further to 2.9% at 4,000 mg/kg Pb^2+^ and to 5.2% at 8,000 mg/kg Pb^2+^ as well as to 6.5% at 16,000 mg/kg Pb^2+^.

### 3.4 Enhancement mechanism

Compared to the immobilization efficiency higher than 90% at 20 mM Pb^2+^, it dropped dramatically to way below 30% at 60 mM Pb^2+^, meaning that the effect of Pb^2+^ toxicity at 60 mM Pb^2+^ was not as less significant as 20 mM Pb^2+^. The urease lost its activity, leading to an inability to catalyze urea hydrolysis and also to secure Pb immobilization. In addition to addressing the inability to provide nucleation sites by the urease, the nHAP application also prevented Pb^2+^ attachment to the urease. The immobilization efficiency, therefore, remained above 70% when even subjected to 60 mM Pb^2+^. C and O elements over a broad range of the SEM-EDS images indicated that under EICP, carbonate minerals contributed primarily to Pb immobilization. Carbonates characterized by strong bands at 1,400 cm^−1^ and in the 678–838 cm^−1^ region provided a strong piece of evidence to support the above claim. On the other hand, Ca and P elements over a broad range of the SEM-EDS images told that under EICP-nHAP, hydroxyapatite was crucial in Pb immobilization. Phosphates specified by strong bands in the 567–603 cm^−1^ region verified the presence of hydroxyapatite (Gao et al., 2019). As discussed above, the nHAP application also adsorbed a part of Pb^2+^ by competing with urease, preventing Pb^2+^ attachment to the urease. This further explained that Pb element distribution under EICP-nHAP was not as even as EICP. Furthermore, the nHAP application was accompanied by Ca^2+^/Pb^2+^ ion exchange (Hopwood et al., 2016). Hydroxylphosphohedyphane or hydroxylpyromorphite at 4.096 Å gave eloquent testimony to support the presence of Ca^2+^/Pb^2+^ ion exchange. Noted that the former was developed when Ca^2+^ was partially replaced with Pb^2+^. Apart from that, the hydroxyl (-OH) in hydroxylpyromorphite was replaced by CO_3_^2−^ at 20 mM Pb^2+^, forming carbonate-bearing hydroxylpyromorphite at typically 3.045 Å (Kwasniak-Kominek et al., 2017). It featured Ksp = 3.98 × 10^−85^ lower than Ksp = 7.4 × 10^−14^ of the cerussite (often seen under EICP), indicating higher chemical stability. This was not the case when subjected to 60 mM Pb^2+^. Hydroxylphosphohedyphane or hydroxylpyromorphite, when subjected to 60 mM Pb^2+^, was formed, followed by cerussite. This allowed hydroxylphosphohedyphane or hydroxylpyromorphite to be wrapped by cerussite, reducing the mobility of Pb^2+^. This explained why cerussite appeared more frequently than hydroxylphosphohedyphane or hydroxylpyromorphite.

On the other hand, the function of nHAP was verified through the stretching vibrations of its functional groups. Although carbonates characterized by strong bands at 1,400 cm^−1^ and in the 678–838 cm^−1^ region were also present under EICP-nHAP, they were not as significant as EICP. Phosphates specified in the 567–603 cm^−1^ region appeared under EICP-nHAP. The nHAP application also caused a shift from 1,050 cm^−1^ to 1,035 cm^−1^. These results gave testimony supporting the claim that the nHAP application prevented Pb^2+^ attachment to the urease and enhanced Pb immobilization. Last but not the least, the good compatibility of nHAP encouraged forming aggregates with the urease, as indicated by the SEM images. The aggregates were applied for biomineralization as nucleation sites. This explained why the nucleated minerals featured stability higher than non-nucleated minerals. The advantages attained under EICP-nHAP elevated the immobilization efficiency to fall within a range of 70%–100%. The horizon of application of this approach is proven rather broad despite the neglection of other influencing factors (e.g., salt pollution).

## 4 Conclusion

The present work compared the performance in Pb immobilization between EICP and EICP-nHAP, and the results accentuated the relative merits of EICP-nHAP. Based on the results and discussion, some main conclusions can be drawn as follows: (ASTM D2487-17, 2010)

(1) The urease lost its activity when Pb^2+^ concentration was in excess of 20 mL/L and therefore, the immobilization efficiency dropped to a value as low as about 25%. nHAP application prevented the urease from being threatened by the effect of Pb^2+^ toxicity, thereby securing the degree of urea hydrolysis. As a result, the immobilization efficiency remained at above 70% when even subjected to 60 mM Pb^2+^.
(2) In addition to addressing the inability to provide nucleation sites by the urease, the nHAP application also prevented Pb^2+^ attachment to the urease. Hydroxylphosphohedyphane and hydroxylpyromorphite provided a strong piece of evidence to support the presence of Ca^2+^/Pb^2+^ ion exchange. The carbonate-bearing minerals were formed at 20 mM Pb^2+^ and featured with stability higher than cerussite (Ksp = 3.98 × 10^−85^ vs. Ksp = 7.4 × 10^−14^). Hydroxylphosphohedyphane or hydroxylpyromorphite +was wrapped by cerussite at 60 mM Pb^2+^, reducing the mobility of Pb^2+^.
(3) The good compatibility of nHAP encouraged forming aggregates with the urease. The aggregates were applied for biomineralization as nucleation sites. This explained why the nucleated minerals featured higher stability. As a result, the advantages attained under EICP-nHAP elevated the immobilization efficiency to fall within a range of 70%–100%.
(4) CAR-Pb was the major species under EICP, while OX-Pb was the major species under EICP-nHAP. Both EICP and EICP-nHAP reduced the fraction of EXC-Pb. OX-Pb was featured with higher stability and lower ecotoxicity than CAR-Pb.

## Data Availability

The original contributions presented in the study are included in the article/Supplementary Material, further inquiries can be directed to the corresponding author.
